# Blood Dendritic Cell Frequency Declines in Idiopathic Parkinson’s Disease and Is Associated with Motor Symptom Severity

**DOI:** 10.1371/journal.pone.0065352

**Published:** 2013-06-11

**Authors:** Antonio Ciaramella, Francesca Salani, Federica Bizzoni, Francesco E. Pontieri, Alessandro Stefani, Mariangela Pierantozzi, Francesca Assogna, Carlo Caltagirone, Gianfranco Spalletta, Paola Bossù

**Affiliations:** 1 Clinical and Behavioral Neurology, IRCCS Fondazione Santa Lucia, Rome, Italy; 2 Department of Neurology and Psychiatry, University “Sapienza”, Movement Disorder Unit, Sant’Andrea Hospital, Rome, Italy; 3 Department of System Medicine, University of “Tor Vergata”, Rome, Italy; Imperial College London, United Kingdom

## Abstract

The role of inflammation in Parkinson’s Disease (PD) is well appreciated, but its underlying mechanisms are still unclear. Our objective was to determine whether dendritic cells (DC), a unique type of migratory immune cells that regulate immunological response and inflammation have an impact on PD. In a case-control study including 80 PD patients and 80 age- and gender-matched healthy control subjects, the two main blood subsets of plasmacytoid and myeloid DC were defined by flow cytometry analysis. Clinical evaluation of subjects consisting of cognition and depression assessment was performed using the Mini Mental State Examination and the Beck Depression Inventory. The severity of motor symptoms was measured using the Unified Parkinson’s Disease Rating Scale-Part III. Comparison between patient and control DC measures and their relationships with clinical assessments were evaluated.The following main results were obtained: 1) the level of circulating DC (mainly the myeloid subset) was significantly reduced in PD patients in comparison with healthy controls; 2) after controlling for depressive and cognitive characteristics, the frequency of myeloid DC was confirmed as one of the independent determinants of PD; 3) the number of both myeloid and plasmacytoid DC was negatively associated with motor symptom severity. Overall, the decline of blood DC, perhaps due to the recruitment of immune cells to the site of disease-specific lesions, can be considered a clue of the immune alteration that characterizes PD, suggesting innovative exploitations of DC monitoring as a clinically significant tool for PD treatment. Indeed, this study suggests that reduced peripheral blood DC are a pathologically-relevant factor of PD and also displays the urgency to better understand DC role in PD for unraveling the immune system contribution to disease progression and thus favoring the development of innovative therapies ideally based on immunomodulation.

## Introduction

Neuroinflammation plays an important role in the pathogenesis of Parkinson’s Disease (PD) [1], but its underlying mechanisms are not well defined. Brain autoantigens like α-synuclein, the main constituent of Lewy bodies, or neuromelanin, another protein derived from the destruction of dopaminergic cells, have been proposed to initiate innate and adaptive immune responses promoting PD neuroinflammatory processes [2], [3]. Inflammation is crucially regulated by dendritic cells (DC), which orchestrate immune responses, leading to the induction of tolerance or immunity [4]. DC can circulate in peripheral blood as patrolling precursors. After having recognized molecules associated with inflammation or tissue damage, including autoantigens, DC migrate to sites of inflammation and to draining lymph nodes where they have the unique ability to educate naïve T cells, connecting innate and adaptive immune responses. A growing body of evidence substantiate the participation of DC in several brain disturbances characterized by neurodegeneration, such as prion disease [5], Alzheimer's disease [6], multiple sclerosis [7], and stroke [8]. Albeit no data on DC involvement in PD pathogenesis were published so far, we hypothesized that DC could play a role in PD by fueling neuroinflammation, since they might be recruited from blood to brain, where initiate the autoantigen driven T cell priming process, and consequently modulate disease progression.

Thus, in the present study, in consideration of the unfeasibility to directly detect DC in the human living brain, we characterized the circulating counterpart of DC in the blood of PD patients. Specifically, by means of flow cytometry analysis, we measured in the whole blood of 80 PD patients, as compared to 80 healthy control (HC) subjects, the frequency of blood DC, corresponding to HLA-DR positive cells, negative for markers of other leukocyte lineages. More in detail, we have taken into consideration the two major subsets of blood DC, namely CD123+ plasmacytoid (pDC) and CD11c+ myeloid (mDC) cells, as they have been widely described in humans before [9], [10]. The frequency of circulating DC was found decreased in patients *versus* matched controls (in particular the mDC subset) and negatively associated with the severity of the disease, ultimately substantiating the main hypothesis of this study that DC may participate in PD pathophysiology.

## Materials and Methods

### Study Population

Blood samples from 80 PD patients and 80 healthy controls (HC) were analyzed.

The diagnosis of idiopathic PD was established according to international guidelines [11]. All the PD patients were recruited at the outpatient Parkinson’s Clinics of the “Sapienza” and “Tor Vergata” Universities of Rome and were assessed at the Neuropsychiatry Laboratory of the IRCCS Fondazione Santa Lucia in Rome. Exclusion criteria for recruitment in the study were the following: 1) history of neurologic diseases other than idiopathic PD; 2) unclear history of dopaminergic treatment responsiveness; 3) presence of major medical illnesses, including overt infectious or auto-immune diseases; 4) treatment with anti-inflammatory or immunosuppressive medication; 5) known or suspected history of alcoholism, drug dependence and abuse, head trauma, or major psychiatric disorders according to the DSM-IV-TR criteria [12], apart from minor depressive disorder; 6) presence of vascular brain lesions or marked cortical and subcortical atrophy based on visual inspection of all clinical MRI sequences by one trained neuroradiologist; 7) dementia diagnosis based on clinical examination or Mini-Mental State Examination (MMSE) [13]; and 8) insufficient vision and hearing to comply with the testing procedure.

Eighty healthy control (HC) subjects, matched with PD patients as for age, education, and gender, also participated in the study (Table 1). Exclusion criteria were the same as for patients with PD, with the exception of points 2 and point 1 modified as: history of neurologic diseases.

**Table 1:** Socio-demographic and clinical characteristics of HC and PD subjects.

Characteristics		HC (n = 80)	PD (n = 80)	*p* value
Gender (men %)		50%	50%	–
Age (years)		59.5±9.5	59.2±9.9	0.833
Education (years )		12.3±3.8	12.6±3.7	0.645
MMSE (score)		29.2±1.2	28.5±2.1	**0.0122**
BDI total (score)		5.3±4.3	10.1±8.0	**<0.0001**
BDI PSY (score)		3.1±2.9	5.6±5.2	**0.0003**
BDI SOM (score)		2.2±2.0	4.5±3.3	**<0.0001**
Disease duration (years)		–	4.6±3.8	–
Hoehn & Yahr (%)	stage 1	–	25%	–
	stage 1.5	–	25%	–
	stage 2	–	32.9%	–
	stage 2.5	–	11.8%	–
	stage 3	–	5.3%	–
	stage >3	–	0%	–
Hoehn & Yahr stage (score)		–	1.74±0.57	–
UPDRS III (score)		–	17.4±9.5	–
L-dopa (mg/day)		–	263.2±287.1	–
DA-LED (mg/day)		–	202.0±271.0	–
Total LED (mg/day)		–	465.2±389.8	–

Values are expressed as mean ± SD or percentage of subjects (%).

The MMSE was used for evaluation of global cognitive performance. Beck Depression Inventory (BDI) was administered to measure severity of depression. In particular, we considered both the psychic (PSY) and somatic (SOM) subscores of these scales in order to distinguish the possible differences between the two dimensions of depression [14].

The Unified Parkinson's Disease Rating Scale–Part III (UPDRS III) [15] was used for the clinical evaluation of motor symptoms.

Hoehn & Yahr score, also used to define disease severity [16], ranged in the enrolled PD subjects between stages 1 and 3, with 50% of patients falling between stages 2 and 3 and a mean score ± SD of 1.74±0.57. At the moment of the clinical assessment and blood sampling collection, less than one third of PD patients (n = 25, 31.25%) were treated with psychotropic drugs, such as antipsychotics (n = 1, 1.25%), antidepressants (n = 11, 13.75%), benzodiazepines (n = 7, 8.75%) or a combination of them (n = 6, 7.5%).

Regarding the dopaminergic therapy of PD patients, 22 (27.5%) were treated with Levodopa (L-dopa), 25 (31.25%) were treated with dopamine agonists (DA) and 26 (32.5%) were treated with both, while 7 (8.75%) were drug naïve. The daily dose of L**-**dopa, as well as the L**-**dopa equivalent dose (LED) used in this study is reported in **Table 1**, where the demographic and clinical characteristics of included subjects are also summarized.

The study was approved by the Fondazione Santa Lucia Ethical Committee and, in accordance with the Helsinki Declaration, each subject signed an informed consent form prior to enrollment.

### Dendritic Cell Characterization

**Figure 1:**
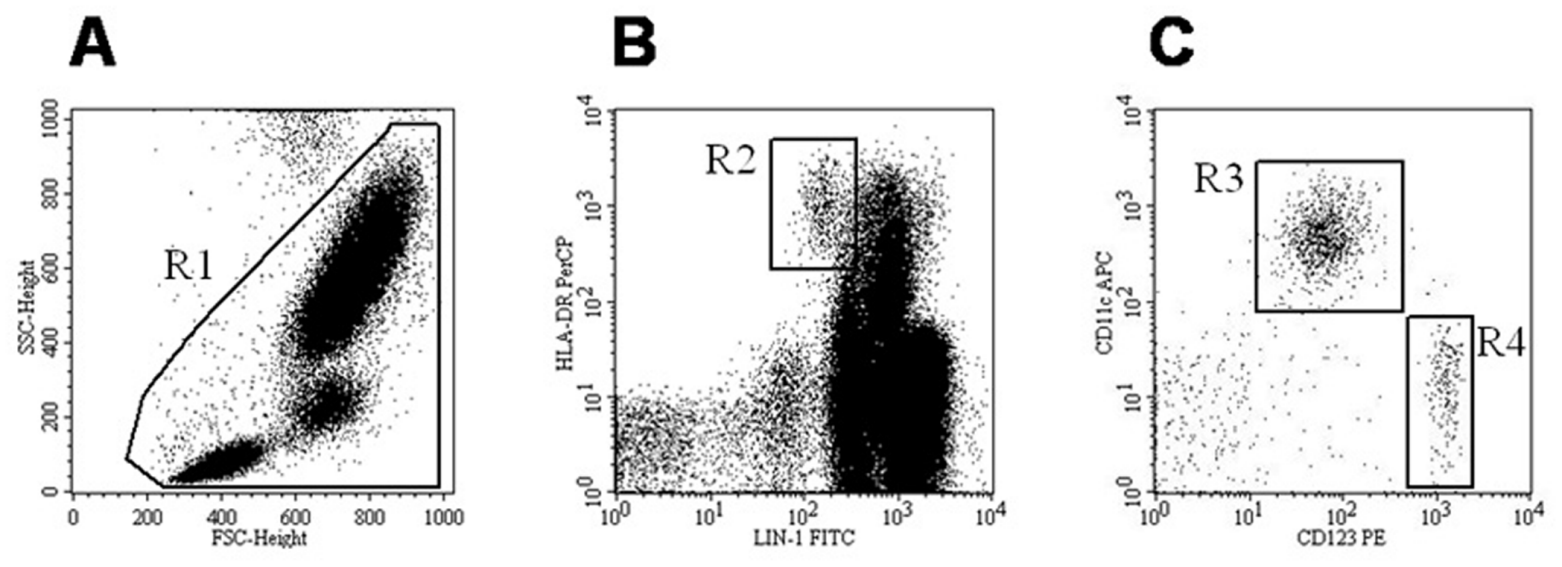
Gating strategy. Representative immunophenotype plots of the whole blood sample from one HC subject are reported to show the gating strategy. Total cells were identified in a forward scatter (FSC)/side scatter (SSC) plot and gated (R1, left panel A). Then DC were identified by gating lineage negative (Lin^−^) cells (R2, middle panel B). Finally, myeloid DC (R3) were identified by CD11c and HLA-DR expression, while plasmacytoid DC (R4) were identified by CD123 and HLA-DR expression (right panel C).

The frequency of peripheral blood DC was assessed by flow cytometry analysis on fresh whole blood within 4 h after sampling. Briefly, 200 µL of heparinized blood was used for each staining tube. Blood cells were incubated for 20 minutes at room temperature with FITC-anti-lineage Cocktail 1 (Lin1, a mix of CD3, CD14, CD16, CD19, CD20, and CD56), PerCP-HLA-DR, APC-CD11c, and PE-CD123. All antibodies were obtained from Becton Dickinson (San Jose, CA). Red cell lysis was performed by the addition of 2 mL of FACS Lysing solution (Becton Dickinson) for 10 minutes according to manufacturer’s instructions. After washing, cells were analyzed in the four-color FACScalibur flow cytometer (BD Biosciences) using the CellQuest software (BD Biosciences). The percentage of the two main subsets of blood DC was defined after gating on the basis of forward and side scatter characteristics, to exclude debris and most polynuclear cells and 5×10^4^ events were analyzed. Then a selection of lineage negative (Lin1^−^) cells was performed. Among these Lin1^−^ cells, pDCs and mDCs were identified as CD123^+^/HLA -DR^+^ and CD11c^+^/HLA-DR^+^, respectively. Representative panels of flow cytometry analysis and gating strategy are reported in **Figure 1**.

DC absolute cell numbers (cells/µL), calculated using DC percentages and total white blood cell (WBC) counts were also reported, as indicated.

### Statistical Analysis

MMSE and BDI scores are reported as mean ± SD and their comparisons between PD and HC groups were analyzed with Student’s t test.

Due to the non parametrical distribution of the DC experimental values, as assessed by Shapiro-Wilk normality test, their frequencies are reported as median with interquartile range and their difference between PD patients and HC subjects were tested using the Mann-Whitney U-test for unpaired data.

Subsequently, In order to identify DC (i.e., pDC and mDC percentages) and clinical (i.e., BDI-PSY, BDI-SOM, and MMSE scores) variables significantly differentiating PD cases and HC, a multivariate logistic regression modelling was used to estimate the odds of independent variables that differed significantly at a p<0.05 level, between PD and HC (considered as dependent variable). The multivariate model was chosen in order to minimize the likelihood of type-I (false positive results) errors. Pre-selection of independent variables to include in the multivariate logistic regression model was done by using Student t-test and U-test analyses in order to determine the significance of differences between PD patients and HC. In the multivariate logistic model only variables with p<0.05 in the pre-selection analyses were included.

Associations between continuous variables were analyzed by nonparametric Spearman rho (ρ) correlation coefficient test. A *p*-value of less than 0.05 was considered statistically significant.

## Results

### Blood DC Decline in PD Patients

In order to measure circulating DC in PD and HC subjects, pDC and mDC subsets were phenotypically analyzed by multiparametric flow cytometry on whole blood, as previously described in details. Since flow cytometry data are expressed as relative percentage values and thus may be open to interpretation depending on total WBC number, DC absolute counts per blood volume were also determined and hereafter shown. For the best characterization of DC distribution, both the measures have been included.

**Figure 2:**
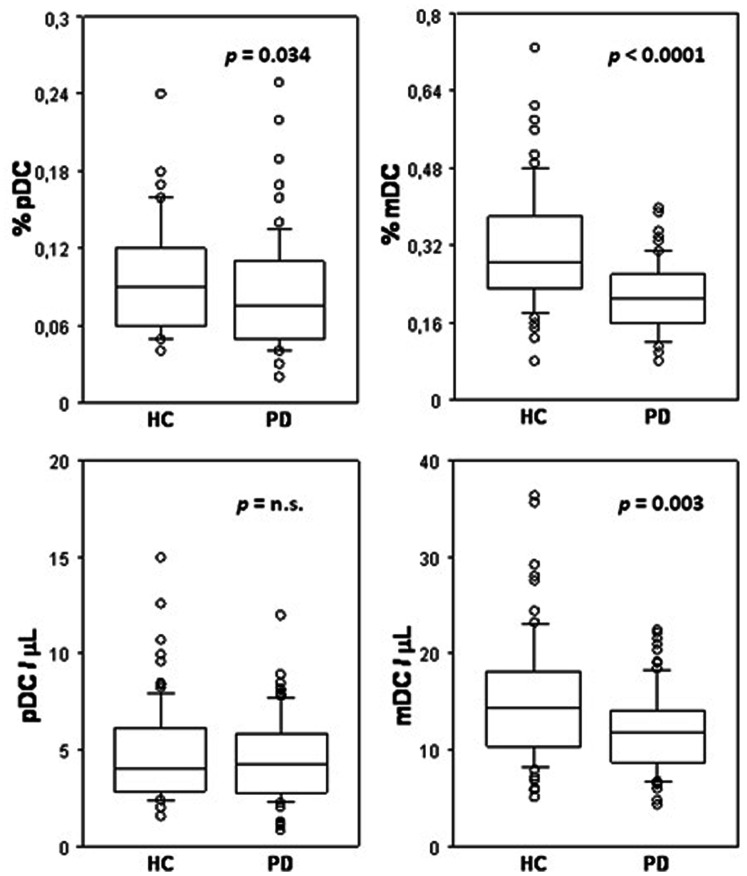
Blood DC decline in PD. The distribution of percentage values (upper panels) and total counts (lower panels) of pDC (left panels) and mDC (right panels) present in blood of HC (n = 80) and PD (n = 80) subjects is reported in the respective box-and-whisker plot, as indicated.

Both relative and absolute measures of DC amount in PD and HC subjects are reported in **Figure 2**. In particular, pDC percentage was decreased in PD patients *versus* HC in a weak but significant manner (U = 2578; *p* = 0.0338), while no significant difference between PD and HC was observed for absolute pDC counts. Differently, mDC frequency was more strongly and significantly reduced in PD patients, as compared to HC, since a statistically significant decrease in mDC percentage (U = 1631; *p*<0.0001) and counts (U = 2326; *p* = 0.0029) was established.

In the multivariate logistic regression, in which all the DC and clinical variables that individually differed between PD and HC were considered as independent variable, the mDC percentage [Odds Ratio (OR) = 14844; 95% Confidence Interval (CI) = 103–2136172; p = 0.0002] was a significant predictor of diagnosis. Specifically, the odds of belonging to the HC group explained 25,1% of the variance (R^2^) of the dependent variable in association with somatic depression (OR = 0.75, 95%CI = 0.62–0.92; p = 0.0067) and global cognitive performance (OR = 1.33, 95%CI = 1.01–1.76; p = 0.0408) that also appeared as independent predictors of belonging to the HC group.

**Figure 3:**
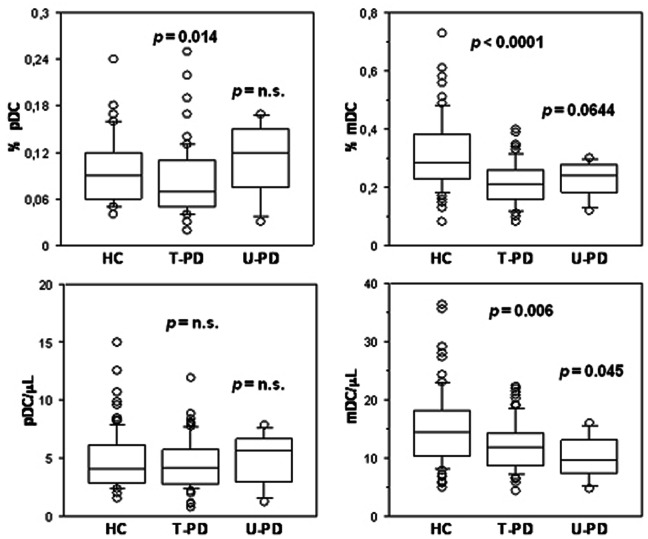
Blood DC decline in PD subgroups. The distribution of percentage values (upper panels) and total counts (lower panels) of pDC (left panels) and mDC (right panels) present in blood of HC (n = 80), dopaminergic drug-treated PD (T-PD, n = 73) and drug naïve PD (U-PD, n = 7) subjects is reported in the respective box-and-whisker plot, as indicated.

Successively, to face the possibility that dopaminergic therapy could play a modulatory effect on DC level, the PD group has been divided into two subgroups, including patients exposed to L-dopa and DA therapy (treated patients, T-PD, n = 73) and the drug naïve patients enrolled in this study (untreated patients, U-PD, n = 7). Similarly to what reported in the whole PD group, a consistent and significant depletion of mDC subsets was observed in both T-PD and U-PD patients *versus* HC, as shown in **Figure 3**. In particular, with regard to cell counts both U-PD (U = 145; *p* = 0.045) and T-PD (U = 2174; *p* = 0.006) differed from HC. Consistently, also mDC percentage of T-PD patients differed from that of HC (U = 1469; *p*<0.0001), and the mDC percentage difference between U-PD patients and HC approached statistical significance (U = 161.5; *p* = 0.0644). As for the pDC subset, cell counts were not significantly different in both PD subgroups as compared with HC. Finally, while the pDC percentage was decreased (U = 2247; *p* = 0.014) in T-PD as compared to HC, it was not different between U-PD and HC.

### DC Frequency, Age and Dopaminergic Therapy

Given that some age-dependent changes in blood DC frequency have been previously reported in non pathological conditions, we evaluated the correlation existing between age and frequency of DC in both HC and PD subjects. A significant negative correlation was found in HC subjects between age and pDC percentage (ρ = −0.411, *p* = 0.0003) or counts (ρ = −0.458, *p*<0.0001), while in PD patients no significant relationships were observed between age and both pDC percentage (ρ = −0.149, *p* = 0.185) and counts (ρ = −0.1, *p* = 0.373). Regarding the mDC subset, a weaker correlation was found in HC as for percentage (ρ = −0.217, *p* = 0.0538) and counts (ρ = −0.270, *p* = 0.0164), with results only approaching the statistical significance in the first case, while in PD patients, no age effect was again reported on both percentage (ρ = −0.192, *p* = 0.0873) and counts (ρ = −0.186, *p* = 0.1) of mDC. Furthermore, DC measures were not affected by gender in both PD and HC individuals (not shown).

**Figure 4:**
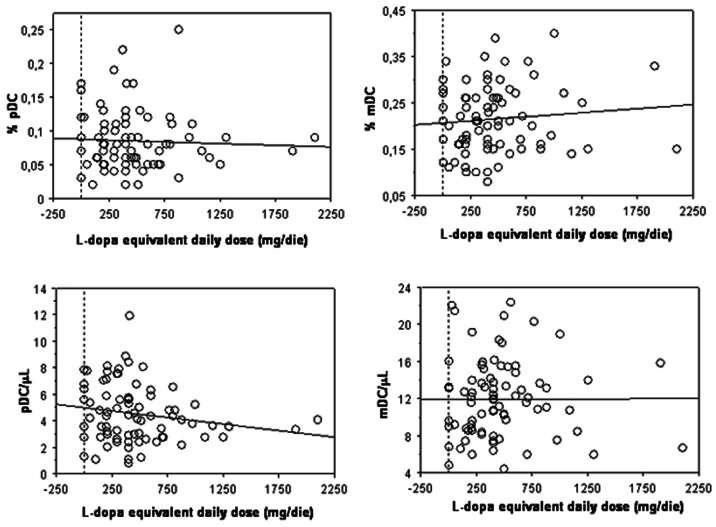
Lack of association between blood DC levels and LED in PD. The correlation of L-dopa equivalent dose with percentage values (upper panels) and total counts (lower panels) of pDC (left panels) and mDC (right panels) present in blood of PD (n = 80) subjects is reported in the respective scatter plot, as indicated. Lines represent linear regression.

Moreover, as shown in **Figure 4**, no significant relationships were found in PD patients between daily LED and mDC percentages (ρ = 0.099 *p* = 0.38) or counts (ρ = 0.109, *p* = 0.331), as well as pDC percentages (ρ = −0.064, *p* = 0.57) or counts (ρ = −0.178, *p* = 0.114).

Similarly, the distribution of DC subsets in PD patients, in terms of either percentage and absolute number, was not affected by disease duration (not shown).

### Impact of DC Frequency on Disease Symptoms

**Figure 5:**
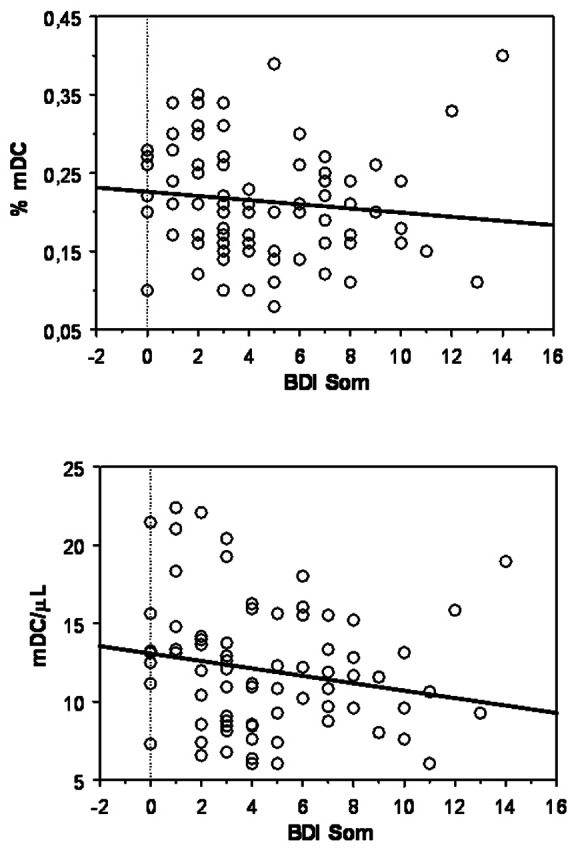
Blood DC association with somatic depression severity in PD. The correlation of BDI SOM scores with percentage values (upper panel) and total counts (lower panel) of mDC present in blood of PD (n = 80) subjects is reported in the respective scatter plot, as indicated. Lines represent linear regression.

To evaluate the impact of blood DC on disease severity, the depressive, cognitive, and motor symptoms were considered. While in PD patients both BDI total and BDI PSY scores were not significantly related to DC measures, a weak but significant negative correlation was found between BDI SOM scores and mDC percentages (ρ = −0.246, *p* = 0.0291) and counts (ρ = −0.225, *p* = 0.0451), as shown in **Figure 5**. Differently, no association was found between any BDI score and blood DC measures in HC (not shown).

Regarding cognitive performances, no significant association was observed between all DC variables and cognitive decline (MMSE score) in both groups (not shown).

**Figure 6:**
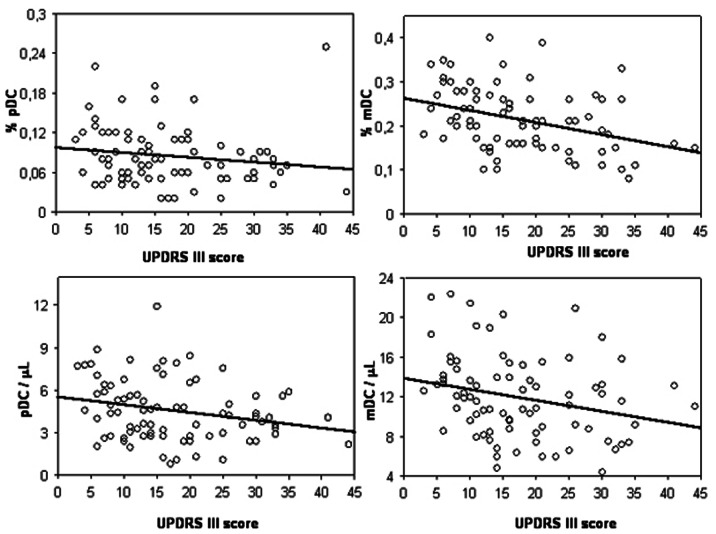
Blood DC association with motor symptom severity in PD. The correlation of UPDRS III score with percentage values (upper panels) and total counts (lower panels) of pDC (left panels) and mDC (right panels) present in blood of PD patients (n = 80) is reported in the respective scatter plot, as indicated. Lines represent linear regression.

Finally, an interesting association between motor symptom severity and amount of circulating DC was observed. In fact, in PD patients the UPDRS III score was negatively related to the percentage of pDC (ρ = −0.223, *p* = 0.0471) and, much more strongly, to the percentage of mDC (ρ = −0.384, *p* = 0.0006). Similar results were also obtained in terms of DC number, with negative significant correlations in both pDC (ρ = −0.245, *p* = 0.0297) and mDC (ρ = −0.295, *p* = 0.0088) subset populations, as reported in **Figure 6**.

## Discussion

Some important results arise from the current flow cytometry evaluation of DC in the whole blood of PD patients. The first finding of this study demonstrates a consistent decline in both percentage and number of mDC in PD patients, as compared to HC. Differently, although percentage of pDC is significantly lower in PD patients in comparison with HC subjects, the pDC number is eventually comparable between the two groups. The discrepancy described between relative and absolute measures of pDC levels in PD patients *versus* HC confirms that changes in pDC subset level, possibly due to the influence of unknown interfering factors that would account for blood cell number fluctuations, appear less suitable than those in mDC, as potential determinant of PD. Indeed, different morphology and functions have been ascribed to the two main pDC and mDC subsets reported in human blood. In particular, pDC appear to differentiate into cells with the typical morphology and functions of DC and are major producers of type I interferon. In contrast, mDC that are often referred to as conventional DCs, may be either a pool of precursors able to migrate into the tissues to refill the DC population, or a sensor of blood borne pathogens, or both [17], [18]. Interestingly, some functional differences in the migratory routes between the two subsets indicate that mDC are more strictly involved in inflammatory processes than pDC [19]. Hence, mDC may be subject to undergo mobilization during PD neuroinflammation more than pDC, ultimately resulting a good candidate to detect PD pathogenic pathways. Importantly, the power of blood mDC decline as predictor of PD has been confirmed in this study by multiple logistic analysis. Thus, although the potential biomarker nature of blood mDC distribution should be confirmed and further investigated, this result holds promises of delivering a new blood-based predictor of disease that probably reflects reactions to the ongoing neurodegenerative processes in PD.

It has been previously reported that DC express dopamine receptors and dopamine can regulate DC functions [20], [21], thus the anti-PD therapy, which is based on either dopamine replacement or dopamine agonist administration, might interfere with circulating DC blood levels. Indeed, our data regarding mDC decrease suggest that this may not be the case. In fact, we have observed both a mDC decline comparable between dopaminergic drug treated and drug naïve patients, and a lack of correlative relationships between LED and DC measures. However, further studies specifically designed to better address this issue should be performed.

Some authors have previously hypothesized that the frequency occurrence of DC subpopulations in the blood might reflect the potential changes in DC homeostasis and trafficking that are associated with the physiopathological state of the individual [10]. Accordingly, blood DC (mainly the pDC subset) appear to decrease with age [22], possibly in relation to the increased susceptibility to infection and decreased response to vaccination typical of aged individuals. In agreement with these published data, we selectively observed in HC subjects an inverse correlation between age and DC frequency (stronger in pDC than mDC subset), while this association was completely skewed in PD patients, strengthening the concept of a general DC perturbation associated with the disease.

With reference to the observation that a pauperization of blood mDC may be linked to the disease state, it should be reminded that in a number of different illnesses, such as viral hepatitis [23], HIV infection [24], tuberculosis [25], or more characteristically, stroke [8], a decline in blood DC, accompanied by local DC increase into inflamed or lymphoid tissues, is described. Therefore, the concept that the decrease in circulating DC may be caused by DC enhanced recruitment at the injury site could be extrapolated also to PD neurodegeneration. Indeed, a number of data indirectly suggest that DC, by virtue of their ability to connect the innate and adaptive arms of the immune system, are pivotal in the PD pathogenesis, where an immune response to self antigens may be a causative factor [26], [27]. In addition, it has been previously proposed that blood-derived DC, which can infiltrate the brain during neuroinflammation, may play some role in neurodegeneration [28].

Since not only DC, but also other peripheral immune system cells are able to infiltrate the brain during neurodegeneration [29], the levels of other white blood cell populations may be altered and relevant in PD progression. Indeed, a modification in the differentiation of hematopoietic stem cells has been described in the peripheral blood of PD patients, with monocyte precursors increased in comparison with controls. Furthermore, similarly to our finding, the percentage of a definite subset of monocytes, which may be in all likelihood recruited to injured brain, is specifically down regulated in the periphery of PD patients as possible consequence of the immune response to an ongoing inflammatory process of the brain [30].

In full accordance with the potential involvement of DC in PD, the second important finding of this study is that in PD patients the frequency of both blood DC subsets are inversely correlated with the decline in motor functions, as measured by UPDRS III scores. Differently from the consistent decrease in DC frequency observed in PD patients, which apparently regards more specifically the mDC subset, the correlation between cell levels and motor symptom severity appears to occur both for mDC and pDC subsets, suggesting that the two types of blood DC may be equally linked to PD evolution, further sustaining the existence of an association between the perturbation of DC homeostasis and the progression of the disease. Such apparent discrepancy could be the general result of different interfering parameters linked to the complexity of both the biological pathways of DC homeostasis and the clinical nature of PD syndrome. More specifically, the effect of factors which are related to the outcome of the disease, rather than to its pathogenesis can participate at DC level to the observed correlations. Hence, PD patients with higher UPDRS III scores may have lower amounts of both DC subsets as a consequence of the immune dysregulation caused by illnesses, secondarily dependent from the severity of PD outcome.

Beyond cardinal motor symptoms, PD course is often complicated by cognitive impairment and by behavioral and psychiatric symptoms and several evidences show that inflammation might contribute to the development of also non-motor symptoms of PD, such as cognitive deficit and depression [31], [32]. In line with this observations, we confirmed that cognitive impairment and depression (particularly somatic depression) are more severe in PD patients, as compared with HC. However, only a significant correlation was observed in PD patients between non-motor symptoms and DC measures, since BDI SOM scores negatively correlated with mDC amounts. Thus, this association supports the view that those depressive symptoms that are more linked to disease neurological aspects or that reflect a reaction to a chronic disabling condition, are possibly related to neuroinflammation through processes that would preferentially involve the myeloid subset of DC. Concordantly, somatic depression, which has a significant impact in PD [33], is associated with inflammation and cell-mediated immune activation [34].

On the whole, we showed here that DC levels decrease as PD specific symptoms (motor dysfunction and somatic depression) become more severe, suggesting that these cells likely represent a biological index of PD progression.

In conclusion, we have provided in the current study the first evidence that peripheral blood DC can be viewed as a new class of pathologically-relevant cells in PD. Thus, even though the mechanisms underlying decline of circulating DC in PD, as well as DC association with disease severity, are not yet known, our data point to an immune participation in PD and advocate the concept that monitoring DC frequency in the blood of these patients may be clinically useful.
